# The effect of assisted enteral feeding on treatment outcome in dogs with inflammatory protein‐losing enteropathy

**DOI:** 10.1111/jvim.16125

**Published:** 2021-05-01

**Authors:** Lavinia Economu, Yu‐mei Chang, Simon L. Priestnall, Aarti Kathrani

**Affiliations:** ^1^ Royal Veterinary College Hatfield United Kingdom; ^2^ Research Support Office, Royal Veterinary College Hatfield United Kingdom; ^3^ Pathobiology and Population Sciences, Royal Veterinary College Hatfield United Kingdom; ^4^ Department of Clinical Science and Services, Royal Veterinary College Hatfield United Kingdom

**Keywords:** canine, intestinal, malnutrition, survival

## Abstract

**Background:**

The effect of assisted enteral feeding on treatment outcome in dogs with protein‐losing enteropathy (PLE) is unknown.

**Objectives:**

To determine if dogs with inflammatory PLE that had an enteral feeding tube placed had better outcome vs dogs with inflammatory PLE without a feeding tube.

**Animals:**

Fifty‐seven dogs with inflammatory PLE.

**Methods:**

A retrospective study at a UK referral hospital identified dogs with inflammatory PLE using a standard diagnostic criterion. Positive outcome was defined as survival greater than 6 months or death unrelated to PLE and negative outcome as death related to PLE within 6 months of diagnosis. Several variables were assessed to identify factors for positive outcome using logistic regression.

**Results:**

Thirty‐five (61%) and 22 (39%) dogs had a positive and negative outcome at 6 months, respectively. Of the 21 dogs that had a feeding tube placed within 5 days of gastrointestinal biopsy, 16 (76%) had a positive outcome and 5 (24%) had a negative outcome. Dogs treated with dietary treatment alone (*P* = .002) and dogs with an enteral feeding tube (*P* = .006) were significantly associated with a positive outcome. When stratified by treatment, assisted enteral feeding was significantly associated with a positive outcome in dogs treated with concurrent immunosuppressive treatment (*P* = .006), but there was insufficient data to evaluate dogs treated with dietary treatment alone.

**Conclusions and Clinical Importance:**

Assisted enteral feeding in dogs with inflammatory PLE could be associated with improved treatment outcome, especially in those receiving immunosuppressive treatment, and should be considered in the treatment plan of these dogs.

**Abbreviations**
BCS: body condition score
CCECAI: canine chronic enteropathy clinical activity index
CI: confidence interval
GI: gastrointestinal
IBD: inflammatory bowel disease
OR: odds ratio
PLE: protein‐losing enteropathy

## 1 INTRODUCTION

The prognosis for inflammatory protein‐losing enteropathy (PLE) in dogs is guarded with disease‐associated death occurring in 54.2% of dogs with this condition.^1^ To date, research on PLE in dogs has predominately focused on determining negative prognostic indicators, with studies identifying serum albumin, bodyweight, serum blood urea nitrogen, and vitamin D metabolites as potential indicators,^2^, ^3^, ^4^, ^5^, ^6^, ^7^ whereas comparatively fewer studies have assessed treatment.^8^, ^9^, ^10^

Although the histopathology of inflammatory bowel disease (IBD) is different between humans and dogs, parallels can be drawn with the treatment approach for both species.^11^ Dietary treatment is an important component in the management of IBD in humans, with studies showing diet can downregulate mucosal pro‐inflammatory cytokines, reduce antigenic stimulation, and reduce prevalence of invasive gastrointestinal (GI) bacterial species.^12^, ^13^, ^14^ Furthermore, enteral feeding is important in human patients with IBD, with use of nasogastric tubes improving remission rates compared to patients administered the dietary treatment PO.^15^ Similarly, dietary treatment is important in dogs with PLE,^1^, ^2^, ^9^ with dogs that are responsive to dietary treatment alone having improved clinical outcomes and increased survival times when compared to dogs receiving immunosuppressive treatment.^5^, ^9^ However, the effects of assisted enteral feeding in dogs with PLE have not yet been assessed.

Anorexia and hyporexia occur in almost 50% of dogs with PLE due to chronic enteropathy and lymphangiectasia.^7^, ^16^ In human patients, anorexia can lead to suppressed crypt cell proliferation, marked decrease in GI‐associated lymphoid tissue and function, increased intestinal permeability, and increased risk of bacterial translocation.^17^, ^18^, ^19^ Dogs with PLE that are anorexic or hyporexic might be at risk of malnutrition, which is associated with numerous detrimental effects in humans, such as immunosuppression, delayed wound healing, loss of muscle mass, and increased in‐hospital mortality and a longer duration of hospital stay in IBD patients.^20^, ^21^, ^22^, ^23^ Therefore, as anorexia and malnutrition might be associated with a weakened immune system and increased risk of intestinal bacterial translocation, commencing glucocorticoids in these dogs might worsen their response to treatment. In contrast, promoting nutritional intake and addressing malnutrition through assisted feeding might result in a stronger enterocyte and immune function. This might lead to an optimal response to immunosuppressive treatment, thereby improving the treatment outcome of dogs with inflammatory PLE.

The aim of our study was to determine whether dogs with inflammatory PLE that had an enteral feeding tube placed at around the time of diagnosis had a better outcome to treatment at 6 months compared to dogs with inflammatory PLE that did not have an enteral feeding tube placed. Age, neutering status, sex, serum albumin and vitamin B12 concentrations, body condition score (BCS), appetite, canine chronic enteropathy clinical activity index (CCECAI), type of dietary treatment, treatment (whether the dogs received dietary treatment alone or immunosuppressive treatment combined with dietary treatment), and year of diagnosis were also evaluated as prognostic indicators for treatment outcome. Our study hypothesized that assisted enteral feeding, initiated at around the time of diagnosis, would improve treatment outcome of dogs with inflammatory PLE at 6 months.

## 2 MATERIALS AND METHODS

### 2.1 Data collection

The electronic medical records of dogs referred to a teaching hospital in the United Kingdom between 2005 and 2020 were reviewed retrospectively to identify dogs with a clinical diagnosis of inflammatory PLE. The following criteria were required for a clinical diagnosis of inflammatory PLE: (a) Evidence of hypoalbuminemia with serum albumin <28 g/L, (b) history of chronic (>3 weeks) GI clinical signs such as weight loss, vomiting, diarrhea, or decreased appetite, (c) exclusion of extraintestinal GI disease such as protein‐losing nephropathy and hepatic disease with diagnostic tests including CBC, serum biochemistry profile, urinalysis, fecal parasitology, abdominal ultrasound, basal cortisol or ACTH stimulation test, vitamin B12 and folate, trypsin like immunoreactivity, and canine pancreatic lipase immunoreactivity, (d) histopathologic confirmation of inflammatory GI disease, defined as a greater increase in lymphocytes, plasma cells or both lymphocytes and plasma cells, in combination with villous architectural changes, or the presence of any number of neutrophils. The final histopathologic diagnosis was based on the predominant inflammatory cell type. All intestinal biopsy specimens and histopathologic diagnoses were reviewed and assigned by a board‐certified veterinary pathologist. Cases with histopathologic confirmation of neoplastic GI disease were excluded.

The following information was collected and evaluated as risk factors from dogs included in the study at the time of histologic diagnosis.

#### 2.1.1 Age, sex, and neuter status

Age (years) was categorized into 4 groups^24^ for analysis: <3, 3 to 5.9, 6 to 8.9, and >9. Sex and neutering status were examined as categorical variables. The breed type was recorded but not included in the risk factor analysis due to inadequate numbers within each breed group.

#### 2.1.2 Assisted enteral feeding

Dogs were categorized into 2 groups based on whether they had received assisted enteral feeding as part of their treatment protocol. Dogs categorized as yes for assisted enteral feeding had a nasogastric, esophagostomy, gastrostomy, or jejunostomy feeding tube placed within 5 days of GI biopsy collection. Dogs categorized as no for assisted enteral feeding did not receive any form of assisted enteral feeding as part of their treatment protocol. The type of feeding tube, the number of days the feeding tube was in place, whether the dog was discharged from the hospital with the feeding tube, and any complications associated with the feeding tube were all recorded for dogs categorized as yes for assisted enteral feeding when the information was available in the clinical notes.

#### 2.1.3 CCECAI scores

The CCECAI scores were used to assess disease severity^25^ and were taken from the clinical notes when written by the clinician; however, when this was not provided, a CCECAI score was calculated retrospectively based on information provided in the history, physical exam, and diagnostic test results. When the required information was not available to calculate CCECAI, a score was not given. The CCECAI scores were categorized into 4 groups^25^: insignificant (score 0‐10), mild (score 11‐15), moderate (score 16‐20), and severe (score 21‐27).

#### 2.1.4 Appetite scores

Appetite scores were given based on information provided in the clinical notes and was defined as the appetite of the dog based on subjective owner assessment at the time of clinical presentation. Appetite scores were defined into 4 separate categories to include anorexia, hyporexia, normal (unchanged), and polyphagia.

#### 2.1.5 Serum vitamin B12 and albumin concentration

For serum vitamin B12 concentration, the concentration within 1 month of histologic diagnosis was used, provided that the animal had not received vitamin B12 supplementation within 1 month of the diagnostic test. Vitamin B12 concentration (ng/L) was categorized as within the reported reference range (>200), lower than the reference range (<200), and unknown. The serum albumin concentration (g/L) at the time of histologic diagnosis was taken and categorized into 4 groups^25^: <12, 12 to 14, 15 to 19, >20 (reference range, 28‐38 g/L).

#### 2.1.6 BCS score

A BCS score at the time of histologic diagnosis was taken from the clinical notes and categorized into 3 groups of under‐condition, ideal body condition, and over‐condition, based on the 9‐point BCS scale^26^: 1 to 3, 4 to 5, >5.

#### 2.1.7 Type of dietary treatment

The type of dietary treatment used during time of hospitalization was recorded and categorized into 4 groups: limited‐ingredient novel protein, hydrolyzed, other, and unknown. In addition, the type of dietary treatment prescribed after hospitalization was recorded. The amount of food consumed before, during, or after hospitalization was not recorded, as this information was not available in the clinical records for the majority of cases.

### 2.2 Year of diagnosis

Dogs were categorized into 3 groups based on their year of histologic diagnosis: 2005 to 2010, 2011 to 2015, and 2016 to 2020.

#### 2.2.1 Treatment

Treatment was characterized based on whether the dog received dietary treatment alone such as a limited‐ingredient novel protein diet or dietary treatment combined with immunosuppressive drugs, including combination treatment with glucocorticoids, cyclosporine, and azathioprine.

#### 2.2.2 Treatment outcome

The treatment outcome for dogs included in the study was recorded. Treatment outcome was determined using information provided in the electronic medical records. When this information was not available, referring veterinarians were contacted to determine the treatment outcome of the dog. The minimum follow‐up time required after date of diagnosis was 6 months. Date of diagnosis was the date at which a histologic diagnosis of inflammatory GI disease was obtained. Treatment outcome was defined as either negative or positive outcome. Positive outcome were dogs that had a survival time equal to or greater than 6 months or death unrelated to PLE. Negative outcome was defined as death related to PLE less than 6 months after diagnosis. Survival time was defined from the time of PLE histologic diagnosis to death or end of the study, or to the last observation recorded before the animal was lost to follow‐up.

### 2.3 Statistical analysis

For the evaluation of risk factors, data collection, checking, and cleaning were performed in Microsoft Excel (2019) producing 1 record per dog, which was then imported into IBM SPSS (Statistical Product and Service Solutions) version 26 statistical software for analysis. Categorical data were summarized with count and percentage. Median and range were calculated for continuous variables. Statistical analysis using univariable logistic regression was performed to test for associations between variables and positive outcome. Variables that were broadly significant within each of the univariable analyses (*P* ≤ .2) were carried forward for multivariable evaluation. Collinearity was assessed between all variables taken forward for multivariable consideration using either Chi‐square or Fisher's exact test. A manual backward stepwise elimination method was used for development of the logistic regression model. Final variables were evaluated for pairwise interaction and statistical significance was set at the 5% level.

Univariable logistic regression was also performed to test for an association between the type of treatment given (dietary treatment alone or immunosuppressive treatment combined with dietary treatment) and CCECAI or serum albumin. In addition, for the dogs that received assisted enteral feeding, a Mann‐Whitney *U* test was used to assess for a significant difference in the number of days the feeding tube was in place and CCECAI or outcome.

## 3 RESULTS

### 3.1 Study sample

#### 3.1.1 Neutering status, sex, and breed

Fifty‐seven dogs met the inclusion criteria: 4 intact females, 28 neutered females, 11 intact males, and 14 neutered males. The study sample had a median age of 6.3 years (range, 0.9‐14.6). Represented breeds are included in the Supporting Information.

#### 3.1.2 Histologic diagnosis

All dogs had small intestinal biopsy specimens collected for histologic diagnosis; 53 dogs (93%) via upper GI endoscopy and 4 (7%) via exploratory laparotomy. All dogs were diagnosed with chronic inflammatory enteropathy; a total of 30 dogs (53%) had lymphoplasmacytic enteritis; 12 (21%) had lymphoplasmacytic enteritis with concurrent lacteal dilatation; 5 (9%) had lymphoplasmacytic and eosinophilic enteritis; 5 (9%) had lymphoplasmacytic and neutrophilic enteritis; 2 (4%) had lymphoplasmacytic, eosinophilic, and neutrophilic enteritis; 1 (2%) had eosinophilic enteritis; 1 (2%) had neutrophilic enteritis; and 1 (2%) had neutrophilic and eosinophilic enteritis.

Colonic biopsy specimens were collected in 24 dogs (42%), 13 (23%) of which also had ileal biopsies performed. These results are included in the Supporting Information.

#### 3.1.3 Serum albumin and vitamin B12 concentration, CCECAI, and BCS

All dogs had hypoalbuminemia (median: 17 g/L; range, 10‐27.7; reference range, 28‐38 g/L). Of the 44 dogs with serum vitamin B12 concentrations available, 28 (64%, 28/44) had concentrations below the reference range. The median for the dogs that had serum vitamin B12 concentrations available was 175 ng/L (range, <150 to 833; reference range, >200 ng/L). The median CCECAI for all dogs was 8 (range, 4‐19) and the median BCS was 3 (range, 1‐8). Eighteen (32%, 18/56) CCECAI scores were calculated retrospectively by 1 of the authors with the remaining 38 (68%, 38/56) calculated by the clinician at the time of diagnosis. There was insufficient information available to calculate the CCECAI retrospectively for 1 dog.

### 3.2 Treatment outcome

Of the 57 dogs with inflammatory PLE, 35 (61%) had a positive outcome and 22 (39%) had a negative outcome. The median survival time for all dogs with PLE was 360 days (range, 0‐3766 days). The median survival time for dogs with a positive outcome was 996 days (range, 180‐3766 days) compared to a median survival time of 12.5 days (range, 0‐174 days) for dogs with a negative outcome.

#### 3.2.1 Age, neutering status, and sex

The median age for the positive outcome group was 6.2 years (range, 1.0‐14.6 years) similar to 6.5 years (range, 0.9‐14.6 years) for the negative outcome group. Neutering status was similar between the 2 groups (Table 1).

**TABLE 1:** Descriptive and univariable logistic regression results for predictors of a positive outcome at 6 months after a histologic diagnosis of inflammatory protein‐losing enteropathy in dogs

Variable	Category	Positive outcome no. (%)	Negative outcome no. (%)	Odds ratio	95% Confidence interval	Variable *P* value
Age (y)	<3	4 (57)	3 (43)	ref		.9
	3‐5.9	11 (61)	7 (39)	1.2	0.2‐6.9	
	6‐8.9	11 (69)	5 (31)	1.7	0.3‐10.3	
	>9	9 (56)	7 (44)	1.0	0.2‐5.8	
Neutering status	Entire	7 (47)	8 (53)	ref		.18
	Neutered	28 (67)	14 (33)	2.2	0.7‐7.6	
Sex	Female	20 (62)	12 (38)	ref		.85
	Male	15 (60)	10 (40)	0.9	0.3‐2.6	
Vitamin B12 (ng/L)^a^	Normal	12 (75)	4 (25)	ref		.34
	Low	17 (61)	11 (39)	0.5	0.1‐2.0	
	Unknown	6 (46)	7 (54)	—	—	
BCS^b^	>3‐5	14 (78)	4 (22)	ref		.22
	1‐3	14 (52)	13 (48)	3.2	0.8‐12.5	
	>5	1 (50)	1 (50)	0.9	0.1‐16.4	
	Unknown	6 (60)	4 (40)	—	—	
Appetite	Normal	12 (67)	6 (33)	ref		.9
	Hyporexia	10 (59)	7 (41)	0.7	0.2‐2.8	
	Anorexia	13 (62)	8 (38)	0.8	0.2‐3.0	
	Unknown	0 (0.0)	1 (100)	—	—	
Albumin (g/L)^c^	>20	13 (68)	6 (32)	ref		.85
	15‐19	15 (56)	12 (44)	0.9	0.1‐12.3	
	12‐14	5 (63)	3 (38)	0.8	0.1‐4.3	
	<12	2 (67)	1 (33)	0.6	0.2‐2.0	
CCECAI^d^	Insignificant	21 (58)	15 (42)	ref		.37
	Mild	5 (50)	5 (50)	0.7	0.2‐2.9	
	Moderate	8 (80)	2 (20)	2.9	0.5‐15.4	
	Unknown	1 (100)	0 (0)	—	—	
Dietary treatment	Novel protein	17 (65)	9 (35)	ref		.43
	Hydrolyzed	14 (78)	4 (22)	1.9	0.5‐7.3	
	Other^e^	3 (50)	3 (50)	0.5	0.1‐3.2	
	Unknown	1 (14)	6 (86)	—	—	
Year of diagnosis	2005‐2010	16 (57)	12 (43)	ref		.74
	2011‐2015	13 (68)	6 (32)	1.6	0.5‐5.5	
	2016‐2020	6 (60)	4 (40)	1.1	0.3‐4.9	
Treatment	Immunosuppressive^f^	19 (48)	21 (53)	ref		.008
	Dietary^g^	16 (94)	1 (6)	17.7	2.1‐146.4	
Enteral feeding^h^	No	19 (53)	17 (47)	ref		.09
	Yes	16 (76)	5 (24)	2.9	0.9‐9.5	

Abbreviations: BCS, body condition score; CCECAI, canine chronic enteropathy clinical activity index; ref, referent.

^a^ Reference range: >200 ng/L.

^b^ Body condition score.

^c^ Reference range: 28‐38 g/L.

^d^ Canine chronic enteropathy activity index; insignificant (0‐10), mild (11‐15), moderate (16‐20), severe (21‐27).

^e^ Other diets refer to therapeutic gastrointestinal diet (n = 3), therapeutic low fat diet (n = 2), or therapeutic high fiber diet (n = 1).

^f^ Dogs that received immunosuppressive treatment combined with dietary treatment.

^g^ Dogs that received dietary treatment alone (without immunosuppressive treatment).

^h^ Dogs categorized into 2 groups based on whether they had received assisted enteral feeding as part of their treatment protocol with the feeding tube placed within 5 days of gastrointestinal biopsy.

#### 3.2.2 Appetite, BCS, CCECAI, serum albumin concentrations, serum vitamin B12 concentrations, and type of dietary treatment

The number of dogs in the positive and negative outcome groups for each of these variables is summarized in Table 1. The median BCS score for the positive outcome group was 3 (range, 2‐8), which was the same for the negative outcome group (range, 1‐5.5). The median CCECAI score for the positive outcome group was 8.5 (range, 4‐19) similar to 8 (range, 4‐17) for the negative outcome group.

Median serum albumin concentration for the positive outcome group was 18.5 g/L (range, 10.0‐27.7; reference range, 28‐38 g/L) compared to 16.0 g/L (range, 10.3‐27.0) for the negative outcome group. Median serum vitamin B12 concentrations were 184 ng/L (range, <150 to 833; reference range, >200 ng/L)) for the positive outcome group, compared to 165 (range, <150 to 593) for the negative outcome group.

All dogs that survived to discharge were prescribed the same diet that they received during hospitalization. Of the dogs included in the “other” category for dietary treatment, 3 received a therapeutic GI diet, 2 received a therapeutic low‐fat diet, and 1 received a therapeutic high fiber diet.

#### 3.2.3 Treatment

For all dogs with PLE, 40 (70%) received immunosuppressive treatment combined with dietary treatment and 17 (30%) received dietary treatment alone. Of the 40 dogs with PLE that received immunosuppressive treatment combined with dietary treatment, 18 (45%) received prednisolone alone, 9 (23%) received prednisolone and cyclosporine, 5 (13%) received cyclosporine alone, 4 (10%) received prednisolone and azathioprine, 2 (5%) received prednisolone and chlorambucil, and 2 (5%) received prednisolone with azathioprine and cyclosporine.

In the positive outcome group, 19 (54%) received immunosuppressive treatment combined with dietary treatment and 16 (46%) received dietary treatment alone. In contrast, the majority of the dogs in the negative outcome group received immunosuppressive treatment combined with dietary treatment (96%, 21), with only 1 dog (5%) receiving dietary treatment alone (Table 1).

#### 3.2.4 Assisted enteral feeding

The study included 21 (37%) dogs that had an assisted enteral feeding tube (esophagostomy tube [19], gastrostomy tube [1], and nasogastric tube [1]). The median number of days the feeding tube was in place was 11 (range, 3‐90 days). However, 3 dogs in the enteral feeding tube group had no information available with regards to when their feeding tube was removed. Two dogs had their assisted enteral feeding tube removed prior to discharge from the hospital due to significant improvement in voluntary food intake. The remaining dogs were discharged from the hospital with their assisted enteral feeding tube in place, except for 1 that died during hospitalization.

Complications associated with the enteral feeding tube were reported for 3 (14%) dogs. The first dog developed purulent discharge at the insertion site on day 6 after esophagostomy tube placement resulting in the feeding tube being removed. In the second dog, the clinician reported mild redness and soreness on the skin around the gastrostomy tube at day 23 after placement; however, the gastrostomy tube was kept in place until day 61. In the third dog, the esophagostomy tube became displaced at day 16 after placement and was subsequently removed. For the 1 dog that had a nasogastric tube, this was in place for 8 days.

For the 21 dogs that received assisted enteral feeding, 14 (67%) had anorexia, with the remaining 7 (33%) being hyporexic. For the 36 dogs that did not receive assisted enteral feeding; 18 (50%) had a normal appetite; 10 (28%) were hyporexic, 7 (19%) were anorexic, and 1 (3%) was unknown.

**FIGURE 1:**
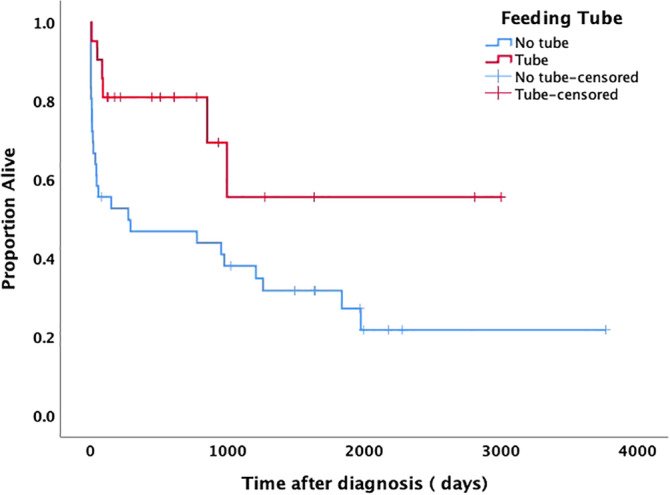
Kaplan‐Meier estimates of survival based on death in dogs with inflammatory protein‐losing enteropathy that received assisted enteral feeding (Tube) compared to dogs that did not receive assisted enteral feeding (No Tube). Marks in the lines indicate censored dogs, which were defined as dogs alive at time of follow‐up

The median survival time for dogs with an assisted enteral feeding tube was 559 days (range, 6‐3149 days), whereas the median survival time for dogs without an assisted enteral feeding tube was 282 days (range, 0‐3766 days; Figure 1).

### 3.3 Statistical analysis

There was no statistically significant association between treatment (whether dogs received dietary treatment alone or immunosuppressive treatment combined with dietary treatment) and CCECAI (*P* = .16) or serum albumin (*P* = .93). In addition, there was no statistically significant difference in the number of days the enteral feeding tube was in place and CCECAI (*P* = .31) or outcome (*P* = .18).

Univariable analysis identified only treatment, whether dogs received immunosuppressive treatment combined with dietary treatment or dietary treatment alone, as a statistically significant variable between positive and negative outcome groups (*P* = .005, Table 1). All other variables were nonsignificant between positive and negative outcome groups (Table 1).

After univariable analysis, the variables retained for multivariable modeling were assisted enteral feeding, treatment, and neutering status. The final multivariable model identified both assisted enteral feeding (*P* = .006) and treatment (*P* = .002) as statistically significant variables between positive outcome and negative outcome groups. Dogs with PLE that received assisted enteral feeding had 7.0 times the odds of a positive outcome (95% confidence interval [CI] = 1.8‐28.2) compared to dogs that did not receive assisted enteral feeding as part of their treatment protocol. At the multivariable level, dogs with PLE that received dietary treatment alone had 36.1 times the odds of a positive outcome (95% CI = 3.9‐333.6) compared to dogs with PLE that received immunosuppressive treatment combined with dietary treatment. There was no significant association between treatment and assisted enteral feeding (*P* = .07). Therefore, the likely reason the multivariable analysis resulted in large changes in odds ratios (ORs) and wide CI for both treatment and assisted enteral feeding is due to only 1 dog in the dietary treatment alone group having a negative outcome.

Therefore, a stratified analysis by treatment was carried out to evaluate the effect of enteral feeding on treatment outcome. For those dogs that received immunosuppressive treatment combined with dietary treatment, assisted enteral feeding increased their odds of a positive outcome by 6.9 times (95% CI = 1.7‐28.0, *P* = .006). For those dogs that received dietary treatment alone, there was insufficient data to evaluate the effects of assisted enteral feeding, as only 1 dog in the dietary treatment alone group had a negative outcome. Also, of the 3 dogs that received dietary treatment alone via assisted enteral feeding, none had a negative outcome, therefore an OR and CI could not be calculated (Table 2).

**TABLE 2:** Stratified logistic regression model with assisted enteral feeding separated by treatment groups (immunosuppressive treatment combined with dietary treatment vs dietary treatment alone) and a positive outcome at 6 months after a histologic diagnosis of inflammatory protein‐losing enteropathy in dogs

Treatment	Enteral feeding^c^	Positive outcome no. (%)	Negative outcome no. (%)	Odds ratio	Confidence interval	*P* value
Immunosuppressive^a^	No	6 (27)	16 (73)	ref		.006
	Yes	13 (72)	5 (28)	6.9	1.7‐28.0	
Dietary^b^	No	13 (93)	1 (7)	ref		1
	Yes	3 (100)	0 (0)	—	—	

Abbreviation: ref, referent.

^a^ Dogs that received immunosuppressive treatment combined with dietary treatment.

^b^ Dogs that received dietary treatment alone (without immunosuppressive treatment).

^c^ Dogs categorized into 2 groups based on whether they had received assisted enteral feeding as part of their treatment protocol with the feeding tube placed within 5 days of gastrointestinal biopsy.

## 4 DISCUSSION

In our study, the use of assisted enteral feeding was significantly associated with a positive outcome in dogs with inflammatory PLE that received immunosuppressive treatment combined with dietary treatment. There was insufficient data to evaluate the effects of assisted enteral feeding in dogs that received dietary treatment alone. In hospitalized dogs and cats, animals that receive 0% to 33% of their calculated maintenance energy requirement are significantly associated with a poorer hospital outcome.^27^ Therefore, ensuring that the dietary requirements of hospitalized dogs with PLE are met via assisted enteral nutrition might have helped to improve their outcome. However, it is still possible that assisted enteral feeding might be less likely to influence the treatment outcome in dogs that receive dietary treatment alone compared to dogs that receive concurrent immunosuppressive treatment. One explanation for this could be that anorexia might be associated with impaired GI mucosal immune function and increased risk of bacterial translocation,^17^, ^18^ administering glucocorticoids in these dogs might worsen their response to treatment. Therefore, promoting nutritional intake via assisted feeding in these dogs might result in a stronger enterocyte and immune function, which might lead to optimal response to immunosuppressive treatment and improved treatment outcome.

In our study, nearly 70% of dogs in the negative outcome group had decreased appetite, which might have limited their nutritional intake of vitamin D, essential amino acids, and other essential nutrients important for gut health. Dogs with chronic enteropathy with moderately/severely decreased appetite have significantly lower serum 25 hydroxyvitamin D (25(OH)D) concentrations than do dogs with chronic enteropathy with normal appetite.^16^ Decreased serum 25(OH) D concentrations are associated with a negative outcome in dogs with PLE^5^ and are associated with increased severity of disease in human IBD patients.^28^ Furthermore, the essential amino acid, tryptophan could play a role in the pathogenesis of PLE in dogs, as serum concentrations are significantly lower in dogs with PLE compared to healthy control dogs.^29^ Although, all dogs on assisted enteral feeding received complete diets, the absolute dietary intake of vitamin D and essential amino acids, such as tryptophan was not evaluated in our study.

The use of enteral feeding tubes in human IBD patients improves compliance to dietary treatment, resulting in higher remission rates compared to patients administered the dietary treatment PO.^15^ Similarly, in our study, assisted enteral feeding might have improved owner compliance to dietary treatment. Adequate owner compliance to dietary treatment is essential, as this might result in an increased likelihood that the dog receives 100% of its daily caloric and nutrient requirement. However, as absolute food intake was not measured or recorded for the dogs at any time point during our study, the effect of enteral feeding on increasing compliance could not be confirmed.

In our study, 17/36 (47%) dogs that did not receive assisted enteral feeding were hyporexic or anorexic. It was not recorded why these dogs did not receive assisted enteral feeding; however, the decision to not place an enteral feeding tube in dogs with PLE with decreased appetite might occur for a number of reasons. For example, owner preference, increased anesthesia time, the assumption that appetite will improve following glucocorticoid treatment,^30^ complicating factors associated with the disease such as coagulopathies^31^ and increased healing time due to hypoalbuminemia.^32^ In our study, the complication rate for dogs with assisted enteral feeding was 14%, compared to 43.1% with esophagostomy tubes.^33^ This could suggest that the complication rate might be underreported in our study. Underreporting might have occurred due to dogs included in our study being lost to follow‐up. For example, dogs receiving assisted enteral feeding might have a greater likelihood in presenting to their referring veterinarian instead of the referral hospital for minor feeding tube complications. However, all feeding tube complications reported in our study were considered as minor and easily manageable, similar to that reported in previous studies.^33^, ^34^ Therefore, our study might be considered to be important in highlighting the benefits of assisted enteral feeding in dogs with inflammatory PLE despite the potential risks involved and should be used to guide the decision making on whether to place an assisted enteral feeding tube in these dogs.

Our study also identified that dogs with PLE that received dietary treatment alone had improved treatment outcome compared to dogs that received immunosuppressive treatment combined with dietary treatment. Dogs with PLE that receive immunosuppressive drugs are significantly associated with a negative outcome.^5^ Also, dogs with PLE that are responsive to dietary treatment alone have longer survival times compared to dogs that require corticosteroid treatment.^9^ One explanation for our results might be that dogs that were responsive to dietary treatment alone were more likely to have a reduced severity of disease compared to dogs that received immunosuppressive treatment, as the CCECAI of dogs that are food‐responsive are significantly lower than dogs that are immunosuppressant‐responsive.^9^ The CCECAI score, which is used as a marker of disease severity, is associated with a negative clinical outcome.^6^, ^7^, ^25^ However, in our study there was no significant association between CCECAI score and serum albumin and whether the dog received dietary treatment alone or immunosuppressive treatment combined with dietary treatment. In addition, there was no significant association between CCECAI and treatment outcome. Therefore, in our study, it is unlikely that lower clinical disease activity was a significant reason for improved treatment outcome in dogs receiving dietary treatment alone. However, a proportion of the CCECAI scores were calculated retrospectively in our study, which might have prevented a significant association from being found between the CCECAI score and treatment or outcome.

Another explanation for our results could be that dogs with PLE that received dietary treatment alone might have had increased intestinal mucosal healing compared to dogs that received concurrent immunosuppressive treatment, thereby improving their treatment outcome. Human IBD patients that receive enteral nutrition (polymeric, semi‐elemental, and elemental diets) have better mucosal healing compared to IBD patients that receive corticosteroid treatment (64.8% vs 40%).^35^ However, prospective studies standardizing clinical disease activity, type and route of dietary treatment and the dose and type of immunosuppressive treatment, with follow‐up intestinal biopsy specimens collected would be needed to confirm this finding.

In addition to the limitations of our study described above, others include the majority of our cases not having ileal biopsies taken; therefore, intestinal neoplasia might have been missed in these dogs, which would have affected the prognosis.^4^ However, lymphoma, the most common primary intestinal neoplasia in dogs, predominately affects the duodenum (85% of dogs) compared to the ileum (55% of dogs).^36^ However, this only applies to small cell lymphoma and does not include other types of intestinal lymphoma or neoplasia. Additionally, histopathologic diagnosis and severity were not evaluated as risk factors in our study, which might have affected the outcome. However, there are no differences in histopathologic grading scores in dogs with chronic enteropathies that are food‐responsive vs steroid‐responsive and no difference in histopathologic grading scores with clinical response to treatment.^37^ Another limitation of our study includes grouping dogs together if they received immunosuppressive treatment regardless of which drug(s) was used, despite dogs with PLE having an improvement in survival times with the use of glucocorticoids alone compared to glucocorticoids combined with a second immunosuppressive agent.^10^ Unfortunately, previous diet history and time to voluntary food intake was not recorded in our study and should be evaluated in future studies. Furthermore, the primary dietary therapies used in our study were novel protein and hydrolyzed diets, with the remaining diets classified into a third “other” category. This might have prevented a statistical significance from being reached between the novel protein and hydrolyzed diets, as dogs with IBD that receive hydrolyzed diets have a greater significant improvement compared to novel protein diets.^38^ In addition, the use of low fat and ultra‐low‐fat diets are associated with good clinical response in dogs with non‐neoplastic PLE.^9^, ^39^ Therefore, standardizing dietary and immunosuppressive treatment as well as the fat content is required for future studies in order to better compare the effect of dietary treatment alone vs combined with immunosuppressive treatment on treatment outcome in dogs with inflammatory PLE. Unfortunately, the type of assisted enteral feeding was not standardized in our study, which might have led to the wide range observed in the duration the feeding tube was in place. Alternatively, dogs with a feeding tube placed for a longer duration might have had a greater severity of disease due to taking a longer time to reach adequate voluntary food intake. However, in our study the number of days the feeding tube was in place was not significantly associated with treatment outcome or CCECAI. A large prospective study that addresses our study limitations is required to definitively assess the effects of assisted enteral feeding on treatment outcome in dogs with inflammatory PLE.

## CONFLICT OF INTEREST DECLARATION

Authors declare no conflict of interest.

## OFF‐LABEL ANTIMICROBIAL DECLARATION

Authors declare no off‐label use of antimicrobials.

## INSTITUTIONAL ANIMAL CARE AND USE COMMITTEE (IACUC) OR OTHER APPROVAL DECLARATION

Approved by the Royal Veterinary College Ethics and Welfare Committee (URN2017 1702‐3).

## HUMAN ETHICS APPROVAL DECLARATION

Authors declare human ethics approval was not needed for this study.

## Supporting information


**Appendix** **S1:** Supporting information

## References

[1] Craven MD , Washabau RJ . Comparative pathophysiology and management of protein‐losing enteropathy. J Vet Intern Med. 2019;33:383‐402. 30762910 10.1111/jvim.15406 PMC6430879

[2] Simmerson SM , Armstrong PJ , Wunschmann A , et al. Clinical features, intestinal histopathology, and outcome in protein‐losing enteropathy in Yorkshire Terrier dogs. J Vet Intern Med. 2014;28:331‐337. 24467282 10.1111/jvim.12291 PMC4857982

[3] Equilino M , Theodoloz V , Gorgas D , et al. Evaluation of serum biochemical marker concentrations and survival time in dogs with protein‐losing enteropathy. J Am Vet Med Assoc. 2015;246:91‐99. 25517330 10.2460/javma.246.1.91

[4] Nakashima K , Hiyoshi S , Ohno K , et al. Prognostic factors in dogs with protein‐losing enteropathy. Vet J. 2015;205:28‐32. 26025135 10.1016/j.tvjl.2015.05.001

[5] Allenspach K , Rizzo J , Jergens AE , et al. Hypovitaminosis D is associated with negative outcome in dogs with protein losing enteropathy: a retrospective study of 43 cases. BMC Vet Res. 2017;13:96. 28390394 10.1186/s12917-017-1022-7 PMC5385077

[6] Gianella P , Lotti U , Bellino C , et al. Clinicopathologic and prognostic factors in short‐ and long‐term surviving dogs with protein‐losing enteropathy. Schweiz Arch Tierheilkd. 2017;159:163‐169. 28248185 10.17236/sat00108

[7] Kathrani A , Sanchez‐Vizcaino F , Hall EJ . Association of chronic enteropathy activity index, blood urea concentration, and risk of death in dogs with protein‐losing enteropathy. J Vet Intern Med. 2019;33:536‐543. 30784115 10.1111/jvim.15448 PMC6430906

[8] Dandrieux JR , Noble PJ , Scase TJ , et al. Comparison of a chlorambucil‐prednisolone combination with an azathioprine‐prednisolone combination for treatment of chronic enteropathy with concurrent protein‐losing enteropathy in dogs: 27 cases (2007‐2010). J Am Vet Med Assoc. 2013;242:1705‐1714. 23725434 10.2460/javma.242.12.1705

[9] Nagata N , Ohta H , Yokoyama N , et al. Clinical characteristics of dogs with food‐responsive protein‐losing enteropathy. J Vet Intern Med. 2020;34:659‐668. 32060974 10.1111/jvim.15720 PMC7096654

[10] Salavati Schmitz S , Gow A , Bommer N , et al. Diagnostic features, treatment, and outcome of dogs with inflammatory protein‐losing enteropathy. J Vet Intern Med. 2019;33:2005‐2013. 31381203 10.1111/jvim.15571 PMC6766500

[11] Cerquetella M , Spaterna A , Laus F , et al. Inflammatory bowel disease in the dog: differences and similarities with humans. World J Gastroenterol. 2010;16:1050‐1056. 20205273 10.3748/wjg.v16.i9.1050 PMC2835779

[12] Griga T , Voigt E , Gretzer B , et al. Increased production of vascular endothelial growth factor by intestinal mucosa of patients with inflammatory bowel disease. Hepatogastroenterology. 1999;46:920‐923. 10370639

[13] Fell JM , Paintin M , Arnaud‐Battandier F , et al. Mucosal healing and a fall in mucosal pro‐inflammatory cytokine mRNA induced by a specific oral polymeric diet in paediatric Crohn's disease. Aliment Pharmacol Ther. 2000;14:281‐289. 10735920 10.1046/j.1365-2036.2000.00707.x

[14] Gerasimidis K , Bertz M , Hanske L , et al. Decline in presumptively protective gut bacterial species and metabolites are paradoxically associated with disease improvement in pediatric Crohn's disease during enteral nutrition. Inflamm Bowel Dis. 2014;20:861‐871. 24651582 10.1097/MIB.0000000000000023

[15] Schwab D , Raithel M , Hahn EG . Enteral nutrition in acute Crohn disease. Z Gastroenterol. 1998;36:983‐995. 9880825

[16] Gow AG , Else R , Evans H , et al. Hypovitaminosis D in dogs with inflammatory bowel disease and hypoalbuminaemia. J Small Anim Pract. 2011;52:411‐418. 21797872 10.1111/j.1748-5827.2011.01082.x

[17] Hernandez G , Velasco N , Wainstein C , et al. Gut mucosal atrophy after a short enteral fasting period in critically ill patients. J Crit Care. 1999;14:73‐77. 10382787 10.1016/s0883-9441(99)90017-5

[18] Deitch EA , Winterton J , Li M , et al. The gut as a portal of entry for bacteremia. Role of protein malnutrition. Ann Surg. 1987;205:681. 3592811 10.1097/00000658-198706000-00010 PMC1493085

[19] Gibson D , Mehler PS . Anorexia nervosa and the immune system—a narrative review. J Clin Med. 2019;8:1915. 10.3390/jcm8111915 PMC6912362 31717370

[20] Detsky AS , Smalley PS , Chang J . The rational clinical examination. Is this patient malnourished? JAMA. 1994;271:54‐58. 8258889 10.1001/jama.271.1.54

[21] Norman K , Kirchner H , Lochs H , et al. Malnutrition affects quality of life in gastroenterology patients. World J Gastroenterol. 2006;12:3380‐3385. 16733855 10.3748/wjg.v12.i21.3385 PMC4087869

[22] Zurita VF , Rawls DE , Dyck WP . Nutritional support in inflammatory bowel disease. Dig Dis. 1995;13:92‐107. 7586636 10.1159/000171491

[23] Nguyen GC , Munsell M , Harris ML . Nationwide prevalence and prognostic significance of clinically diagnosable protein‐calorie malnutrition in hospitalized inflammatory bowel disease patients. Inflamm Bowel Dis. 2008;14:1105‐1111. 18302272 10.1002/ibd.20429

[24] Wang T , Ma J , Hogan AN , et al. Quantitative translation of dog‐to‐human aging by conserved remodeling of the DNA methylome. Cell Syst. 2020;11:176‐185. 32619550 10.1016/j.cels.2020.06.006 PMC7484147

[25] Allenspach K , Wieland B , Grone A , et al. Chronic enteropathies in dogs: evaluation of risk factors for negative outcome. J Vet Intern Med. 2007;21:700‐708. 17708389 10.1892/0891-6640(2007)21[700:ceideo]2.0.co;2

[26] Mawby DI , Bartges JW , d'Avignon A , et al. Comparison of various methods for estimating body fat in dogs. J Am Anim Hosp Assoc. 2004;40:109‐114. 15007045 10.5326/0400109

[27] Brunetto MA , Gomes MO , Andre MR , et al. Effects of nutritional support on hospital outcome in dogs and cats. J Vet Emerg Crit Care. 2010;20:224‐231. 10.1111/j.1476-4431.2009.00507.x 20487250

[28] Tan B , Li P , Lv H , et al. Vitamin D levels and bone metabolism in Chinese adult patients with inflammatory bowel disease. J Dig Dis. 2014;15:116‐123. 24354597 10.1111/1751-2980.12118

[29] Kathrani A , Allenspach K , Fascetti AJ , et al. Alterations in serum amino acid concentrations in dogs with protein‐losing enteropathy. J Vet Intern Med. 2018;32:1026‐1032. 29604114 10.1111/jvim.15116 PMC5980272

[30] Elkholly DA , Brodbelt DC , Church DB , et al. Side effects to systemic glucocorticoid therapy in dogs under primary veterinary care in the UK. Front Vet Sci. 2020;7:515. 32923470 10.3389/fvets.2020.00515 PMC7457010

[31] Goodwin LV , Goggs R , Chan DL , et al. Hypercoagulability in dogs with protein‐losing enteropathy. J Vet Intern Med. 2011;25:273‐277. 21314726 10.1111/j.1939-1676.2011.0683.x

[32] Doweiko JP , Nompleggi DJ . The role of albumin in human physiology and pathophysiology, part III: albumin and disease states. JPEN J Parenter Enteral Nutr. 1991;15:476‐483. 1895489 10.1177/0148607191015004476

[33] Nathanson O , McGonigle K , Michel K , et al. Esophagostomy tube complications in dogs and cats: retrospective review of 225 cases. J Vet Intern Med. 2019;33:2014‐2019. 31294877 10.1111/jvim.15563 PMC6766496

[34] Breheny CR , Boag A , Le Gal A , et al. Esophageal feeding tube placement and the associated complications in 248 cats. J Vet Intern Med. 2019;33:1306‐1314. 31001901 10.1111/jvim.15496 PMC6524112

[35] Berni Canani R , Terrin G , Borrelli O , et al. Short‐ and long‐term therapeutic efficacy of nutritional therapy and corticosteroids in paediatric Crohn's disease. Dig Liver Dis. 2006;38:381‐387. 16301010 10.1016/j.dld.2005.10.005

[36] Couto KM , Moore PF , Zwingenberger AL , et al. Clinical characteristics and outcome in dogs with small cell T‐cell intestinal lymphoma. Vet Comp Oncol. 2018;16:337‐343. 29322604 10.1111/vco.12384 PMC6041184

[37] Schreiner NM , Gaschen F , Grone A , et al. Clinical signs, histology, and CD3‐positive cells before and after treatment of dogs with chronic enteropathies. J Vet Intern Med. 2008;22:1079‐1083. 18673423 10.1111/j.1939-1676.2008.0153.x

[38] Marchesi MC , Timpano CC , Busechian S , et al. The role of diet in managing inflamatory bowel disease affected dogs: a retrospective cohort study on 76 cases. Vet Ital. 2017;53:297‐302. 29307123 10.12834/VetIt.566.2700.1

[39] Rudinsky AJ , Howard JP , Bishop MA , et al. Dietary management of presumptive protein‐losing enteropathy in Yorkshire terriers. J Small Anim Pract. 2017;58:103‐108. 28160309 10.1111/jsap.12625

