# Supplementary material for: The effect of assisted enteral feeding on treatment outcome in dogs with inflammatory protein‐losing enteropathy
Source: J Vet Intern Med. 2021 May 1;35(3):1297–305. doi: 10.1111/jvim.16125 (PMC8163126; doi:10.1111/jvim.16125)
Supplement: Supplementary file 1 — Appendix S1: Supporting information [file JVIM-35-1297-s001.pdf]

Supplementary Material

Breeds represented in the study

Breeds included in the study: Crossbreed (6), Cocker Spaniel (5), Cavalier King Charles Spaniel (5), Labrador (4), Dogue de Bordeaux (3), Border Collie (3), Golden Retriever (3), Greyhound (2), Miniature Schnauzer (2), Weimaraner (2) and one of each of the following breeds: American Bulldog, Basset Fauve, Border Terrier, Boxer, English Bulldog, English Springer Spaniel, French Bulldog, Griffon, Hungarian Vizsla, Irish Setter, Jack Russell Terrier, Kerry Blue Terrier, Miniature Bull Terrier, Miniature Poodle, Rottweiler, Staffordshire Bull Terrier, Schnauzer, Shar-Pei, Standard Poodle, Toy Poodle, Tibetan Terrier and Yorkshire Terrier.

Histopathologic diagnosis of colonic and ileal biopsy specimens

Colonic biopsy specimens were collected in 24 dogs (42.1%), 13 (22.8%) of which also had ileal biopsies performed. Ten (17.5%) of the colonic biopsy specimens were within normal limits; eight (14.0%) had lymphoplasmacytic colitis; three (5.3%) had neutrophilic colitis; one (1.8%) had lymphoplasmacytic and eosinophilic colitis; one (1.8%) had lymphoplasmacytic and neutrophilic colitis and one (1.8%) had lymphoplasmacytic and histiocytic colitis.

Of the 13 dogs that had ileal biopsies taken; five (8.8%) had lymphoplasmacytic ileitis with concurrent lacteal dilation, three (5.3%) had lymphoplasmacytic and neutrophilic ileitis, two (3.5%) had lymphoplasmacytic ileitis and one (1.8%) had eosinophilic ileitis.
